# Effect of straw biochar amendment on tobacco growth, soil properties, and rhizosphere bacterial communities

**DOI:** 10.1038/s41598-021-00168-y

**Published:** 2021-10-20

**Authors:** Jiayu Zheng, Jixu Zhang, Lin Gao, Rui Wang, Jiaming Gao, Yanchen Dai, Wei Li, Guoming Shen, Fanyu Kong, Jiguang Zhang

**Affiliations:** 1 Tobacco Research Institute of Chinese Academy of Agricultural Sciences, Qingdao, 266101 People’s Republic of China; 2 Tobacco Company of Hubei Province, Wuhan, 430030 People’s Republic of China; 3 Kunming Tobacco Company, Kunming, 651500 People’s Republic of China; 4 China Tobacco Jiangsu Industrial Co., Ltd., Nanjing, 210019 People’s Republic of China

**Keywords:** Environmental chemistry, Environmental impact, Microbiology, Environmental sciences

## Abstract

Biochar is an effective soil conditioner. However, we have limited understanding of biochar effects on the tobacco growth and bacterial communities in rhizosphere. The aim of this study was to investigate the effects of different straw biochar amendment (0, 2, 10, and 50 g/kg dry soil) on tobacco growth, soil properties, and bacterial communities in rhizosphere by pot trials. Most of tobacco agronomic traits increased when the application rate varied from 0 to 10 g/kg, but were inhibited by 50 g/kg of biochar application. Soil pH, SOC, available nutrients and soil urease, invertase, and acid phosphatase activities were all increased with the biochar application, whereas catalase activity decreased or remained unchanged. The OTUs and bacterial community diversity indices differed with the biochar application doses in rhizosphere and non-rhizosphere soils. And significant differences in bacterial communities were found between the rhizosphere and non-rhizosphere soils despite the biochar addition. *Firmicutes*, *Proteobacteria*, *Acidobacteria*, *Bacteroidetes*, and *Actinobacteria* were the dominant phyla in all soil samples, but they had different abundances in different treatment influenced by the rhizosphere and biochar effect. The high dose of biochar (50 g/kg) decreased the similarity of soil bacterial community structure in rhizosphere compared with those in non-rhizosphere soil. These results provide a better understanding of the microecological benefits of straw biochar in tobacco ecosystem.

## Introduction

Biochar is a carbon-rich by-product of thermal degradation of organic materials under anaerobic environment (i.e. pyrolysis), and it can be distinguished from charcoal owing to its soil amendment properties^1^. Biochar contains large amounts of carbon and macro- or micro-nutrients, which depends on the feedstock and pyrolysis temperature^2,3^. It can be produced from a wide range of biomass sources, such as woody materials, agricultural waste, animal manure, and other waste products^4–6^. Biochar is gaining recognition from scientists and policy makers for its potential role in carbon sequestration, reduction of greenhouse gas emissions, renewable energy production, waste mitigation, and soil amendment^1^.

Previous studies have reported biochar as a soil conditioner used for enhancing soil fertility and crop productivity in different soils, crops, and agroecosystems^7–9^. The observed effects of biochar on soil fertility are pH adjustment in acid soils and improvement in nutrient retention through cation adsorption^10^. The amendment of agricultural soils with different types of biochar had variable effects on soil properties depending on the soil type, biochar feedstock, and amendment rate^11^. Meanwhile, some studies showed that high biochar addition rate had negative effects on soil quality and crop quantity parameters, such as short-term reduction in soil mineral N availability^4,12^, inhibition of soil microbial biomass and activity^13^, and inhibition of crop yield^14^. Furthermore, biochar addition had different effects on plant disease resistance according to biochar dose and biochar type^15,16^.

Enzymatic assays are a valuable tool for understanding the soil metabolic activity that underpins processes, such as mineralization and humification of soil organic matter. Soil enzymes are catalysts of organic matter decomposition, and they are involved in biogeochemical nutrient cycling^16^, including carbon (C), nitrogen (N), phosphorous (P), and sulfur (S) cycling. Recently, some studies have reported that biochar addition to soil increased the activity of soil enzymes related to N and P cycling and reduced the activity of soil enzymes involved in C cycling^17,18^. Conversely, other studies have obtained inconsistent results^16^, which suggested that biochar has variable effects on different soils, enzymes, and assay types.

Soil physicochemical properties can be affected by biochar application, which changes the habitat for microbial colonization, thereby affecting soil microbial activity and microbial community structure^19,20^. Changes in mic robial communities can be associated with changes in soil nutrients, pH, and physical properties after biochar addition^21,22^. Some studies found that biochar addition could affect soil bacterial and fungal community structure^23,24^. Crop rhizosphere is known to be a hotspot for microbial diversity as well as to contain different microbial communities than bulk soil^25^. Biochar may alter the soil microbial community structure, which can consequently change the physical interactions between plant roots and microorganisms^26^. However, despite its critical importance for nutrient acquisition and soil-borne disease control, the effects of biochar on rhizosphere microbial communities are not well understood and need to be further studied^27^.

Flue-cured tobacco (*Nicotiana tabacum L*.), one of the most important industrial crops, is extensively planted in the south-central region of China. The effect of biochar on soil physicochemical properties, plant growth, and the rhizosphere microbiome of different crops has been studied frequently^7,12,15,19,22^. However, most studies were conducted in bulk or in greenhouses and focused on rhizosphere soil only. Straw biochar, as a soil amendment, began to be applied into tobacco field in recent years, and the related research is still at an early stage. Hence, the aim of this study was to evaluate the effect of biochar addition on tobacco growth, soil nutrients, enzymes, and the rhizosphere bacterial community structure using a pot experiment, and to determine whether the changes in the bacterial community in the rhizosphere and non-rhizosphere soil are consistent after biochar application. We hypothesized that the biochar would have a different effect on rhizosphere bacteria compared with the non-rhizosphere soil, which directly drives the process of nutrient transformation and hence the growth of tobacco.

## Materials and methods

### Site description and experiment description

The pot experiment was conducted in 2017 at the Wangchengpo Modern Tobacco Agricultural Science and Technology Park in Enshi City, Hubei Province, China. The test soil for the experiment was collected from mature tobacco fields in the village of Mao Bacao, Baiguo Town, Hubei Province. The main characteristics of the soil prior to experimentation were as follows: pH 6.9; soil organic carbon, 19.29 g/kg; alkali-hydrolysable N, 86.25 mg/kg; available phosphorus (Olsen-P), 76.15 mg/kg; and extractable K, 292.82 mg/kg. The tested soil was classified as Haplic Luvisol (UNESCO soil classification system) and characterized as a silty loam.

The following five treatments were used in the pot experiment: CK1 (without fertilizer and biochar addition), CK2 (with fertilizer addition and without biochar addition), T1 [fertilizer + 0.2% biochar (2 g/kg soil dry weight) addition], T2 treatment [fertilizer + 1.0% biochar (10 g/kg soil dry weight) addition], and T3 treatment [fertilizer + 5.0% biochar (50 g/kg soil dry weight) addition]. The soil was air-dried, passed through a sieve with 2-mm mesh size, and mixed with chemical fertilizers (4 g N per pot and N: P_2_O_5_: K_2_O = 1:1.5:3, in line with the amount of chemical fertilizer applied in the local tobacco planting area). The amount of chemical fertilizer applied in all treatments (except CK1) was also equivalent to that in the local tobacco field. Biochar was crushed and then passed through a sieve with 2 mm mesh size and mixed with the soil and chemical fertilizers in the pots. Each plastic pot was filled with 15 kg sieved soil, and watered until 60% water holding capacity (WHC) during the experimental period. The treatments were conducted in five replicates each. The tested flue-cured tobacco variety was “Yunyan 87”, and the plants were transplanted to one plant/pot on May 10. Biochar was provided by the Institute of Soil Science, Chinese Academy of Sciences. The biochar was produced from rice straw. The conversion of the rice straw to biochar was accomplished under slow pyrolysis, at a heating rate of 10 ℃/min to a highest temperature of 500 ℃, with a 4 h residence time. The main characteristics of rice straw biochar were as follows: pH 9.2; total carbon, 630 g/kg; total nitrogen, 13.5 g/kg; ash content, 140 g/kg; total phosphorus, 4.50 g/kg; total potassium, 21.5 g/kg; total cadmium, 0.69 mg/kg; total arsenic, 0.78 mg/kg; total lead, 5.60 mg/kg; total chromium, 9.09 mg/kg, and total nickel, 5.71 mg/kg.

The soil that was strongly adhered to the roots and within the area influenced by the roots was considered as rhizosphere soil (R-soil)^28^. R-soil samples were collected from mature tobacco plants on September 5, 2017 by the root-shaking method^29^. Meanwhile, the non-rhizosphere soil (N-soil) samples were collected from five points in each pot using a soil corer, mixed, and homogenized to acquire approximately 0.5 kg of the final samples. Soil samples (200 g) were air-dried and further sieved through a 2 mm mesh size for chemical and enzyme activity analysis. The remaining R-soil (RCK1, RCK2, RT1, RT2, and RT3) and N-soil samples (NCK1, NCK2, NT1, NT2, and NT3) were collected and stored at − 80 °C for further analyses of the soil microbial community.

### Tobacco agronomic traits analysis

Five pots per treatment were used to record the main agronomic trait indices of mature tobacco (September 5, 2017). Tobacco height, stem girth, and number of productive leaves were measured with a tape. Meanwhile, three representative tobacco plants were selected, and their entire root systems were pulled out and rinsed thoroughly with water. The roots, stems, and leaves of tobacco plants were taken as samples. These samples were heated at 105 °C for 30 min and then dried at 65 °C for 72 h to a constant weight followed by the measurement of the biomass of three samples. All methods were performed in accordance with the relevant guidelines and regulations^30^.

### Soil chemical properties

The soil pH was measured electrometrically in distilled water solution at a 1:2.5 (w/v) ratio. Soil organic carbon was determined through the potassium dichromate method. Available N (alkali-hydrolyzable N) was determined by the alkali solution method and available P was determined by using Olsen’s method^31^, which is a colorimetric estimation using ammonium molybdate after extraction of phosphate with sodium bicarbonate. Available K (extractable K) was determined by a flame photometer using ammonium acetate. All the above methods have been described in detail in Sparks et al. (1996)^32^.

### Soil enzyme analysis

Enzymatic activities of sucrase, urease, and acid phosphatase in the soil were measured using the principles of colorimetry. Catalase activity was determined by volumetric titration. Sucrase activity was determined by 3, 5-dinitrosalicylic acid colorimetry using sucrose as the substrate, and it was expressed as mg glucose/g dry soil 24 h^−1^^33^. Soil urease activity was measured using indophenol colorimetry with urea as the substrate. Ammonium ions were released over 1 h and assayed calorimetrically at 578 nm. Soil urease activity was expressed as mg NH^4+^–N/ kg dry soil/h^34^. Soil acid phosphatase activity was determined by phenyl phosphate colorimetry according to Guan (1986)^35^, and catalase activity was determined according to Xu and Zheng (1986)^36^. Acid phosphatase activity was expressed as mg pnitrophenol/g dry soil/d, and catalase activity was expressed as ml of 0.02 N KMnO_4_/g dry soil/min. The above estimations of enzymatic activities were performed in triplicates and their mean values were reported.

### Soil DNA extraction and polymerase chain reaction (PCR) sequencing

The total genome DNA from soil samples was extracted by following the instructions of the Power Soil DNA extraction kit (MOBIO, USA). The integrity of the DNA was determined by 1% agarose gel electrophoresis, while the purity and concentration of the DNA were assessed using Nanodrop microvolume spectrophotometer (Thermo Fisher Scientific, Waltham, MA, USA).

The universal 16S/18S rRNA genes were amplified using barcoded primers (16S V4: 515F-806R, 18S V4: 528F-706R, ITS1: ITS1F- ITS2). All PCR reactions were carried out in 30 μL capacity reactors with 15 μL of Phusion High-Fidelity PCR Master Mix (New England Biolabs); 0.2 μM of forward and reverse primers; and about 10 ng template DNA. Thermal cycling consisted of initial denaturation at 98 °C for 1 min, followed by 30 cycles of denaturation at 98 °C for 10 s, annealing at 50 °C for 30 s, elongation at 72 °C for 60 s, and finally subjection to 72 °C for 5 min. The same volume of 1X loading buffer (containing SYB green) with PCR products were mixed and electrophoresis was initiated on 2% agarose gel for detection. Samples with bright strip between 400 and 450 base pairs (bp) were chosen for further experimentation. PCR products were mixed in ratios of equal density. Later, this mixture was purified with a Gene JET Gel Extraction Kit (Thermo Scientific). Sequencing libraries were generated using NEB Next Ultra DNA Library Prep Kit for Illumina (NEB, USA) following manufacturer’s recommendations, and index codes were added. The library quality was assessed on the Qubit 2.0 Fluorometer (Thermo Scientific) and Agilent Bioanalyzer 2100 system. Finally, the library was sequenced on an Illumina MiSeq platform at Majorbio Bio-Pharm Technology Co., Ltd. (Shanghai, China) and 250 bp/300 bp paired-end reads were generated. Raw reads were deposited in the NCBI Sequence Read Archive (SRA) database. Data were also analysed by Majorbio Bio-Pharm Technology Co., Ltd. (Shanghai, China).

### Pyrosequencing data processing

Sequences were processed using the quantitative insights in microbial ecology (QIIME) pipeline v.1.3.0^37^. After sequence splitting by barcode, pre-processing filtration was done using default settings along with 600 bp maximum sequence length to account for length amplification. The remaining sequences (33.6%) were clustered using uclust^38^ into OTUs (operational taxonomic units) with a 0.03 dissimilarity index (97% sequence similarity). Paired-end reads from the original DNA fragments were merged using FLASH^39^, which has been designed to merge paired-end reads when some of the reads overlap with the read generated from the opposite end of the same DNA fragment. Paired-end reads were assigned to each sample according to the unique barcodes. Sequences analysis was performed by UPARSE software package using the UPARSE-OTU and UPARSE-OUT ref algorithms. In-house Perl scripts were used to analyze alpha (within samples) and beta (among samples) diversity. Sequences with ≥ 97% similarity were assigned to the same OTUs. We picked a representative sequence for each OTU and used the ribosomal database project (RDP) classifier to annotate taxonomic information for each representative sequence. To compute alpha diversity, we refined the OTU table and calculated the following metrics: Chao and Abundance-based Coverage Estimator (ACE) indices for species abundance, observed species index for quantitative estimation of unique OTUs in each sample, and the Shannon and Simpson indices. Graphical representation of the relative abundance of bacterial diversity from the phylum to the gene level can be visualised using a Krona chart. We used the unweighted pair group method with Arithmetic mean (UPGMA) clustering^37^. UPGMA Clustering is a type of hierarchical clustering method using average linkage and can be used to interpret the distance matrix.

### Statistical analysis

In the present study, Shannon's index and Simpson's index (D) were used to estimate the diversity of microbial community. A high value of Shannon's index indicates high diversity; while a low value of D, indicates high diversity. Chao1 and ACE were used to estimate the richness of microbial community, where high values of the two indices indicate high community richness. Phylogenetic distance and the Shannon index were computed in QIIME. Overall, bacterial communities’ structural changes were evaluated by principal coordination analysis (PCoA) with the genescloud tools (https://www.genescloud.cn). Permutational Multivariate Analysis of Variance (PERMANOVA) were used to perform the beta-diversity analysis. The differences in soil properties, soil enzyme activities and alpha diversity of bacterial communities between treatments were tested by One-way Analysis of Variance (ANOVA). The data obtained in the experiment were analysed using SPSS 16.0. (SPSS Inc., Chicago, IL, USA). Multiple comparisons were used to determine the significant difference among the treatments at *p* < 0.05.

## Results

### Effect of biochar on tobacco agronomic traits

The significant difference test results for effect on tobacco growth performance after biochar application is shown in Table 1. Overall, the growth performance of tobacco was the lowest in CK1 treatment. The highest growth performance of tobacco was observed in T2, and which was significantly different from T3, which showed the lowest growth performance among all biochar addition treatments. There were no significant differences between the growth performance of tobacco in T2, T1, and CK2. Additionally, there were no significant differences in the stem girth of tobacco with and without biochar application (*p* > 0.05).

**Table 1:** Effect of straw biochar application on the agronomic traits and biomass of tobacco at mature stage.

Treatments	Plant height (cm)	Stem girth (cm)	Productive leaves number	Root biomass (g)	Stem biomass (g)	Leaves biomass (g)
CK1	86.21c	5.11a	18.31b	8.01b	16.21d	32.21d
CK2	112.00ab	5.37a	21.33a	9.17c	24.53b	45.46ab
T1	115.67ab	5.33a	21.67a	9.70bc	27.89a	48.47a
T2	127.33a	5.83a	21.00ab	10.83b	23.21b	48.67a
T3	103.00b	5.17a	19.33b	11.99a	19.64c	40.21c

Different letters in each column indicate significant differences (*p* < 0.05) among the treatments for each parameter.

The root biomass of tobacco decreased in the order T3 > T2 > T1 > CK2 > CK1; there were significant differences between the root biomasses in T3, T2, and CK2. The stem biomass decreased in the order T1 > CK2 > T2 > T3 > CK1, and a significant difference was observed between the stem biomass in T1 and CK2 as well as between T3 and CK2. The leaf biomass decreased in the order T2 > T1 > CK2 > T3 > CK1, and a significant difference was seen between the leaf biomass in T3, T1, and T2. Based on these results, we can conclude that an appropriate dose of biochar can promote tobacco growth, but higher doses of biochar (50 g/kg) can inhibit tobacco growth.

### Effects of biochar on soil chemical properties

The changes in soil chemical properties in response to biochar application are shown in Table 2. Overall, soil pH, organic carbon, and available nitrogen, phosphorus, and potassium increased with the increase in biochar doses. The highest pH (7.72) was observed in T3, followed by T2, and they were significantly higher than that in other treatments. Soil organic carbon in T3 was significantly (*p* < 0.05) higher than in the other treatments. Available nitrogen and available potassium in soil showed similar trends, and the highest values were both in T3, which were significantly higher than those in other treatments. Available phosphorus in T1 was not significantly different from CK2 and T2. Low dose of biochar addition (2 g/kg) did not significantly improve the soil chemical properties, except for improvement in the available potassium content. However, higher doses of biochar addition (10 g/kg and 50 g/kg) significantly improved the soil chemical properties.

**Table 2:** Effect of straw biochar application on soil chemical properties.

Treatments	pH	Organic carbon (g/kg)	Available nitrogen (mg/kg)	Available phosphorus (mg/kg)	Available potassium (mg/kg)
CK1	6.35c	11.24c	79.82d	58.76d	568.21e
CK2	6.38c	11.39c	92.18c	81.97c	695.49d
T1	6.56c	12.32bc	105.48c	87.40bc	994.16c
T2	7.12b	13.00b	124.09b	92.26b	1373.12b
T3	7.72a	43.92a	140.05a	118.88a	2352.77a

Different letters in each column indicate significant differences (*p* < 0.05) among the treatments for each parameter.

### Effects of biochar on soil enzyme activities

The effects of enzyme activities to biochar application are shown in Table 3. All soil enzyme activities except for catalase activity showed low effects in CK1. Soil urease, invertase, and acid phosphatase activities increased to different extents with the increase in biochar application. Soil urease activity for different treatments was in the order T3 > T1 > T2 > CK2 > CK1, and it increased by 13.16%, 2.63%, and 121.05% in T1, T2, and T3, respectively, compared to CK2. In addition, urease activity was significantly higher in T3 than other treatments. Soil invertase activity was in the order T3 > T2 > T1 > CK2 > CK1, and it increased by 70.59%, 82.35%, and 376.47%, in T1, T2, and T3, respectively, compared to CK2. And the invertase activity was significantly higher in T3 than T1, T2 and CK2. Acid phosphatase activity decreased in the order of T1 > T2 > T3 > CK2 > CK1, and it increased by 40.82%, 34.69%, and 6.12% in T1, T2, and T3, respectively, compared to CK2. There were no significant differences among the three biochar addition treatments. However, catalase activity demonstrated a different trend compared to other enzymes, and it decreased in the order of CK2 = T3 > CK1 > T1 > T2. And the catalase activity in CK2 and T3 was significantly higher than T1 and T2. Thus, low dose of biochar addition (2 g/kg-10 g/kg) may inhibit soil catalase activity.

**Table 3:** Effect of straw biochar application on soil enzyme activities.

Treatments	Urease activities (NH_3_–N mg g^−1 ^d^−1^)	Invertase activities (Glu mg g^−1 ^d^−1^)	Acid phosphatase activities (Phenol mg g^−1 ^d^−1^)	Catalase activities (0.02 mol L^−1^ KMnO_4_ ml g^−1 ^min^−1^)
CK1	0.37 b	0.16 c	0.48 b	0.15 a
CK2	0.38 b	0.17 c	0.49 b	0.16 a
T1	0.43 b	0.29 b	0.69 a	0.08 b
T2	0.39 b	0.31 b	0.66 a	0.07 b
T3	0.84 a	0.81 a	0.52 ab	0.16 a

Different letters in each column indicate significant differences (*p* < 0.05) among the treatments for each parameter.

### Effects of biochar on soil bacterial community structure

Illumina pyrosequencing was used to analyse the bacterial community diversities and phylogenetic structures in treatments with different biochar applications. The soil bacterial community richness (number of observed OTUs, ACE, and Chao1) and diversity (Shannon and Simpson) differed with the amount of biochar (Table 4). The number of observed OTUs in all treatments in N-soils and R-soils was in the order CK1 > T1 > T3 > T2 > CK2 and T2 > T3 > CK2 > T1 > CK1, respectively. The number of observed OTUs, ACE, and Chao1 indices showed a similar trend in the treatments of N-soils or R-soils. The three indices were highest in CK1 and lowest in CK2, and they were higher in the biochar treatments than in CK2 (*p* < 0.05) in N-soils. For the three biochar treatments, there were no differences (*p* > 0.05). Furthermore, the three indices were higher in T2 and T3 than in T1 and CK2 in the R-soils.

**Table 4:** Bacterial community abundance and diversity indices of the 16S rRNA gene libraries for clustering at 97% identity in different treatments.

Treatments	Number of observed OTUs	Ace	Chao1	Shannon	Simpson
NCK1	1512 a	2708 a	2253 a	4.95 ab	0.083 a
NCK2	874 c	1784 c	1434 c	4.79 b	0.062 b
NT1	1476 ab	2027 b	2063 ab	4.86 b	0.066 ab
NT2	1355 b	1924 bc	1878 b	5.16 a	0.044 c
NT3	1470 ab	2066 b	2107 ab	4.90 ab	0.066 ab
RCK1	865 c	1280 c	1220 c	2.90 d	0.265 a
RCK2	1499 b	1890 b	1830 b	5.32 a	0.048 c
RT1	1090 c	1565 bc	1571 bc	3.65 c	0.234 ab
RT2	1734 a	2500 a	2486 a	4.39 b	0.151 b
RT3	1571 ab	2328 a	2246 a	5.12 a	0.081c

RCK1: R-soil in CK1; RCK2: R-soil in CK2; RT1: R-soil in T1; RT2: R-soil in T2; RT3: R-soil in T3; NCK1: N-soil in CK1; NCK2: N-soil in CK2; NT1: N-soil in T1; NT2: N-soil in T2; NT3: N-soil in T3. Different letters in each column indicate significant differences (*p* < 0.05) among the N-soil or R-soil treatments for each parameter.

The Shannon index in N-soils was the highest in T2 followed by CK1, T3, T2, and CK2. The Shannon index in T2 was higher than in CK2 and T1 (*p* < 0.05). There was no significant difference in Shannon index between T2 and T3. Similarly, the Shannon index in T3 was higher than in T1 and T2 (*p* < 0.05) in R-soils, and there were no significant differences in Shannon index between T3 and CK2. The ACE and Chao1 indices showed a similar pattern in R-soils, where the indices were the highest in T2 and lowest in CK1 and were different from each other (*p* < 0.05). Furthermore, the Simpson index of soil bacterial diversity was lowest in T2, which was lower than in other treatments in N-soils. The Simpson index was lowest in CK2 followed by T3, which were lower than in the other treatments (*p* < 0.05).

**Figure 1:**
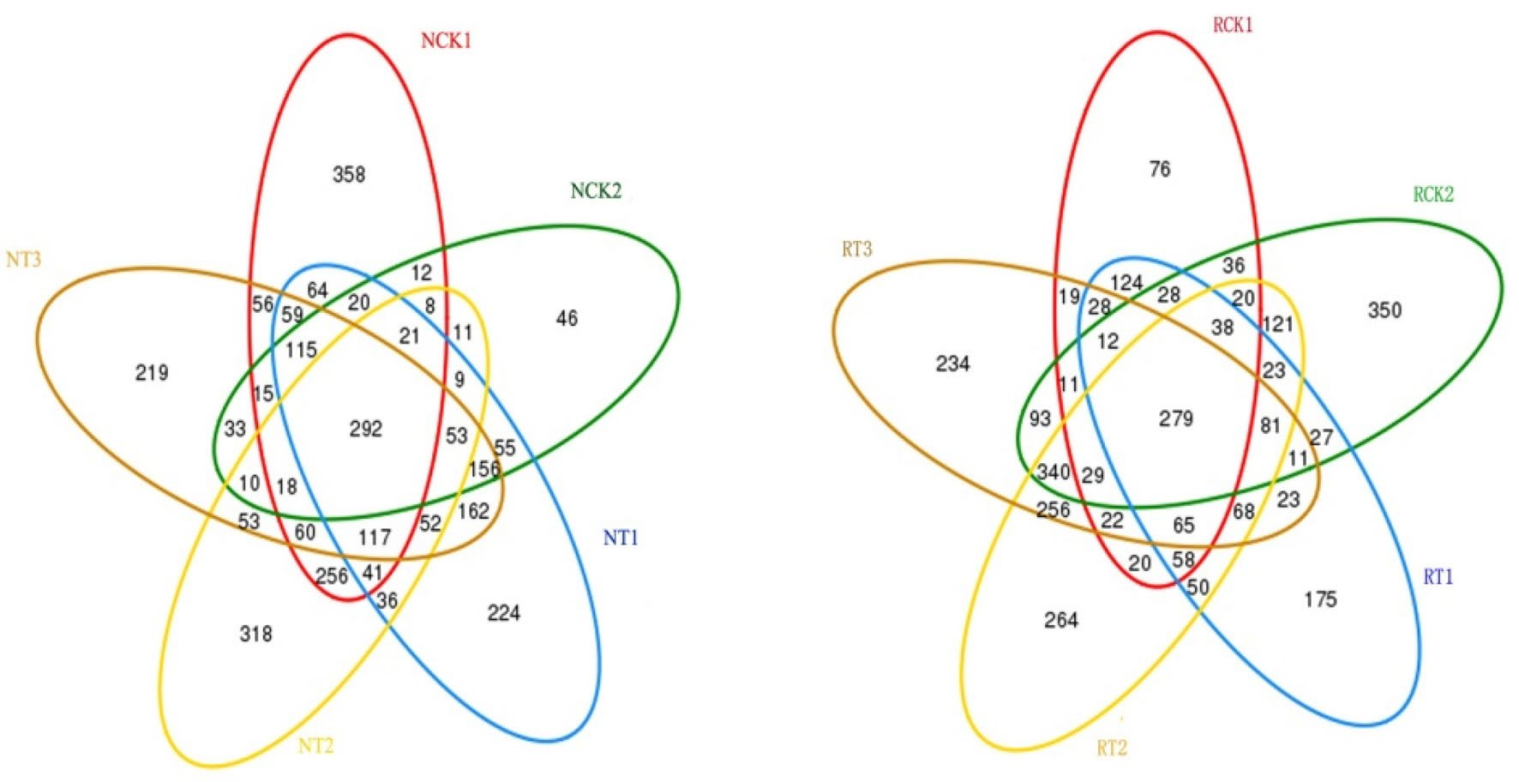
Venn diagrams showing the distribution of OTUs among different treatments. RCK1: R-soil in CK1; RCK2: R-soil in CK2; RT1: R-soil in T1; RT2: R-soil in T2; RT3: R-soil in T3; NCK1: N-soil in CK1; NCK2: N-soil in CK2; NT1: N-soil in T1; NT2: N-soil in T2; NT3: N-soil in T3.

**Figure 2:**
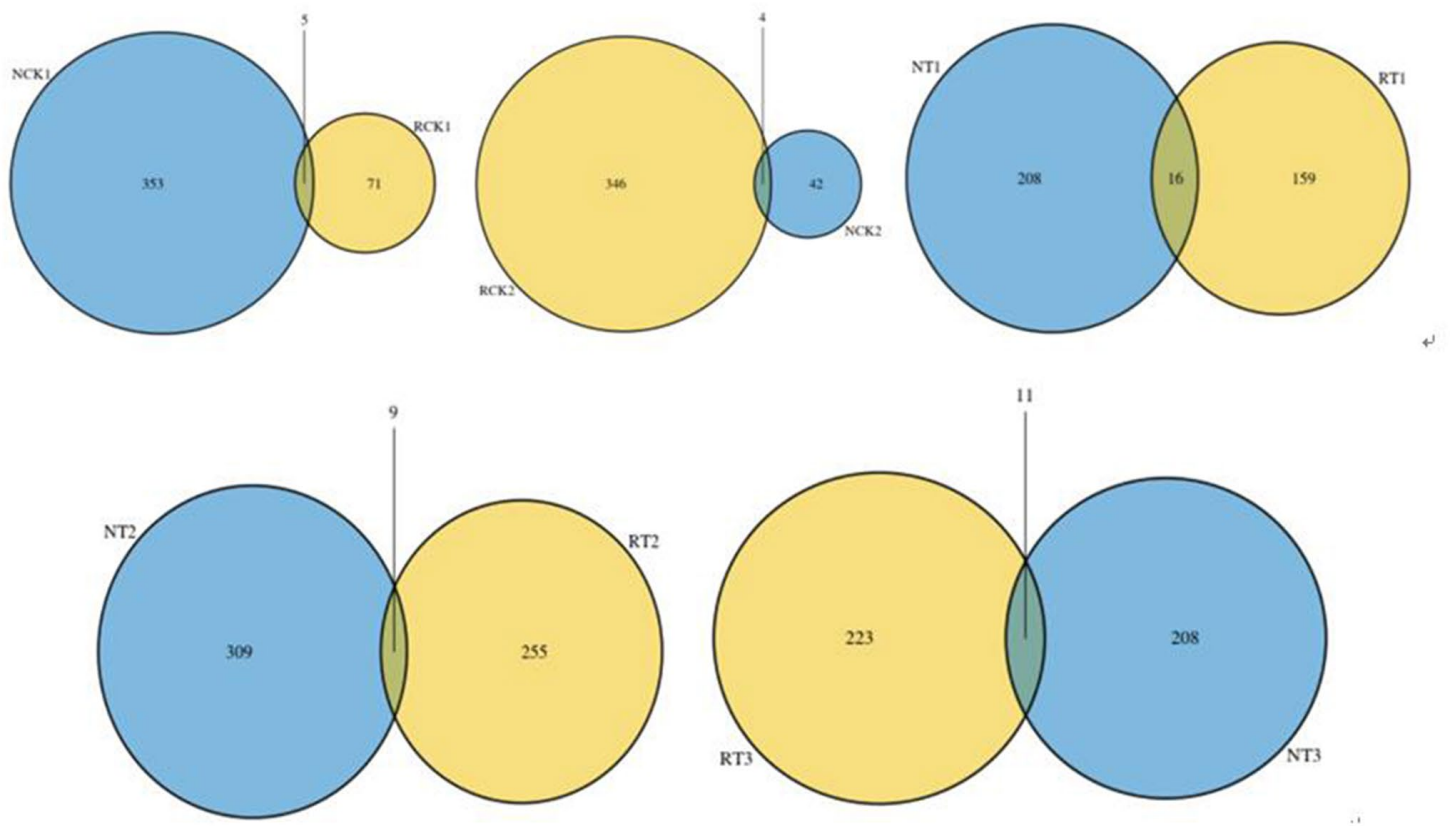
Venn diagrams showing the distribution of OTUs between the rhizosphere soil and bulk soil of different treatments. RCK1: R-soil in CK1; RCK2: R-soil in CK2; RT1: R-soil in T1; RT2: R-soil in T2; RT3: R-soil in T3; NCK1: N-soil in CK1; NCK2: N-soil in CK2; NT1: N-soil in T1; NT2: N-soil in T2; NT3: N-soil in T3.

A Venn diagram (Figs. 1, 2) was used to evaluate the distribution of OTUs among the different treatments. Figure 1 shows that, on average, 292 OTUs containing 9.83% of all sequences were common to the N-soil samples, which comprised 19.31%, 33.41%, 19.78%, 21.55%, and 19.87% of OTUs in NCK1, NCK2, NT1, NT2, and NT3, respectively. Additionally, 279 OTUs containing 9.36% of all sequences were common to the R-soil samples, which comprised 32.25%, 18.61%, 25.60%, 16.09%, and 17.76% of OTUs in RCK1, RCK2, RT1, RT2, and RT3, respectively. Figure 2 illustrates the shared or unique OTUs between the R-soil and N-soil of different treatments. The minimal overlap of OTUs in the figure indicates the great differences in bacterial communities between R-soil and N-soil despite the biochar addition.

**Figure 3:**
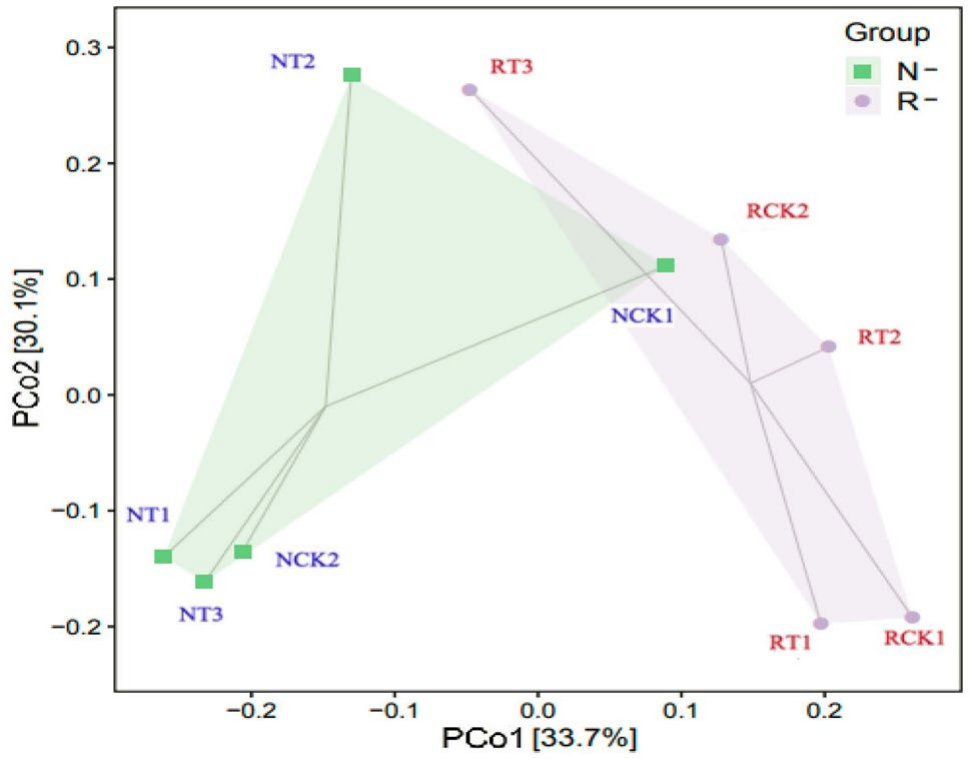
PCoA plot of different soil samples based on Bray–Curtis distance measures. Values on PCoA axes indicate the percentages of total variation explained by each axis and the relative abundances of OUT were used as input in the analysis. RCK1: R-soil in CK1; RCK2: R-soil in CK2; RT1: R-soil in T1; RT2: R-soil in T2; RT3: R-soil in T3; NCK1: N-soil in CK1; NCK2: N-soil in CK2; NT1: N-soil in T1; NT2: N-soil in T2; NT3: N-soil in T3.

Majority of the shared OTUs belonged to *Firmicutes*, *Proteobacteria*, and *Acidobacteria*. The PCoA result evidently suggested the separate bacterial communities in rhizosphere and non-rhizosphere soil samples (Fig. 3). The first axis (PCo1) explained 33.7%, whereas the second axis (PCo2) explained 30.1%. In total, the two axes explained 63.8% of species variance. This indicated that the bacterial communities in R-soil samples were similar and clustered in one group, whereas communities were similar in N-soil samples and also clustered in another group. And the PERMANOVA test based on the Bray–Curtis distance measures showed that the bacterial community structure was significantly (PERMANOVA; *p* = 0.019) different between these two clusters grouped by R-soil and N-soil in all treatments. It was further confirmed that the root exudates and rhizosphere environment had important effects on soil bacterial community structure.

**Figure 4:**
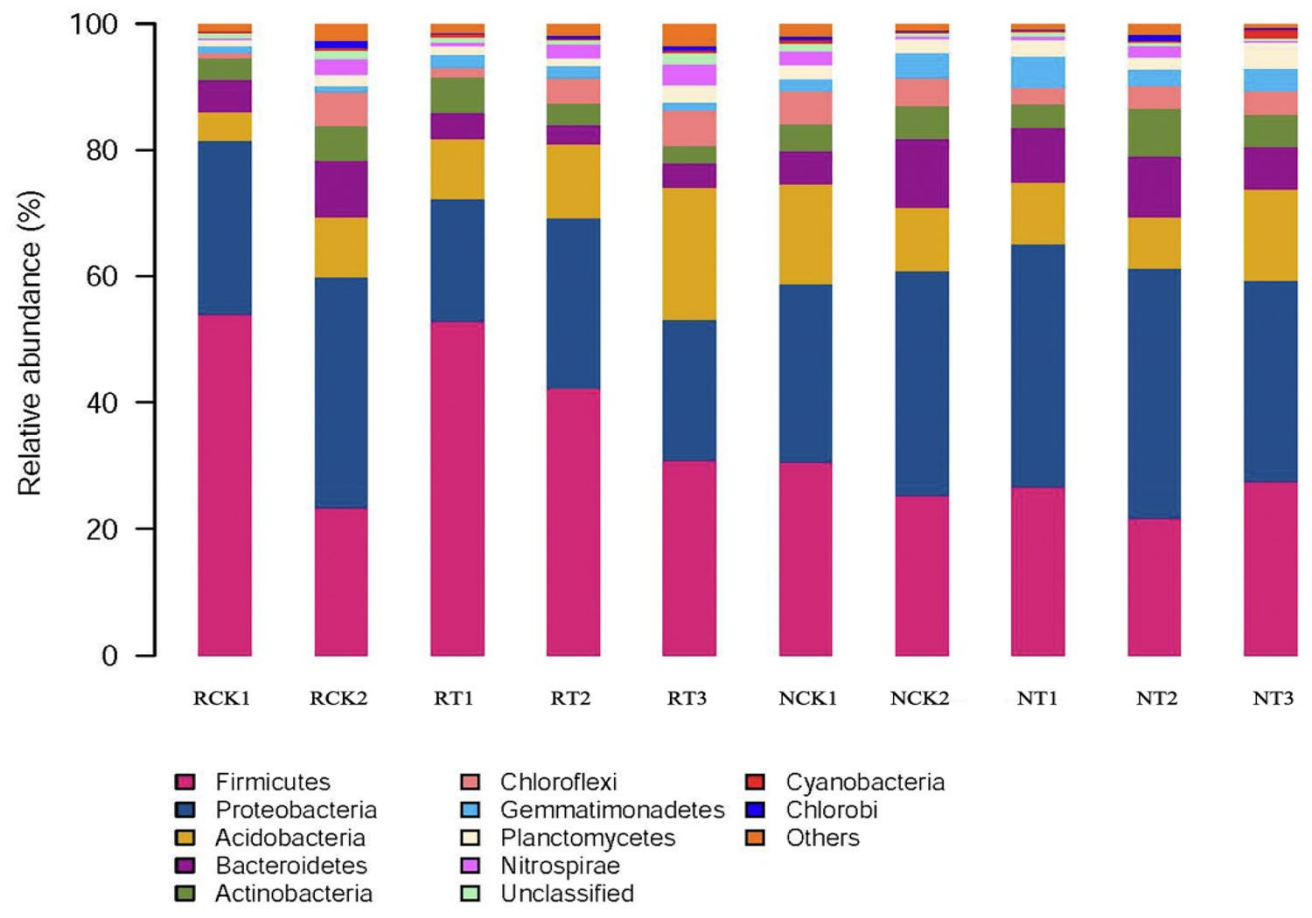
Relative abundance of different bacterial community structures in different treatments at the phylum level. RCK1: R-soil in CK1; RCK2: R-soil in CK2; RT1: R-soil in T1; RT2: R-soil in T2; RT3: R-soil in T3; NCK1: N-soil in CK1; NCK2: N-soil in CK2; NT1: N-soil in T1; NT2: N-soil in T2; NT3: N-soil in T3; N-soil: non-rhizosphere soil (N-soil); R-soil: rhizosphere soil (R-soil); CK1: without fertilizer and biochar addition; CK2: with fertilizer addition and without biochar; T1: fertilizer + 0.2% biochar addition; T2 treatment: fertilizer + 1.0% biochar addition; and T3: fertilizer + 5.0% biochar addition.

The bacteria from all soil samples had similar diversity, but different abundances. The relative bacterial community abundance at the phylum level is illustrated in Fig. 4. In total, 12 phyla were identified, among which *Firmicutes*, *Proteobacteria*, and *Acidobacteria* were the dominant phyla in all soil samples. The relative abundance of different phyla, especially the dominant phyla, differed among different treatments. In the CK2 N-soil sample, *Firmicutes* accounted for 25.34% of the total bacteria, whereas it accounted for 26.61%, 21.74%, and 27.55% of total bacteria in T1, T2, and T3, respectively. *Proteobacteria*, another predominant phylum, accounted for 35.45% of the total bacteria in CK2, and it accounted for comparatively higher proportion in T1 (38.45%) and T2 (39.49%), but comparatively lower proportion in T3 (31.76%). In comparison with the *Acidobacteria* abundance in CK2 (10.02%), the abundance was clearly higher in T3 (14.42%), and lower in T1 (9.87%) and T2 (8.18%).

In the R-soil samples, the abundance of *Firmicutes* in CK2 accounted for 23.39% of the total bacteria at the phylum level, which was evidently lower than T1 (52.86%), T2 (42.26%), and T3 (30.79%). The abundance of *Proteobacteria*, another predominant phylum, accounted for 36.41% of the total bacteria in CK2, which was higher than T1 (19.41%), T2 (27.02%), and T3 (22.38%). The abundance of *Acidobacteria* was high in T2 (11.70%) and T3 (20.87%), and comparatively lower in T1 (9.42%) and CK2 (9.61%).

**Figure 5:**
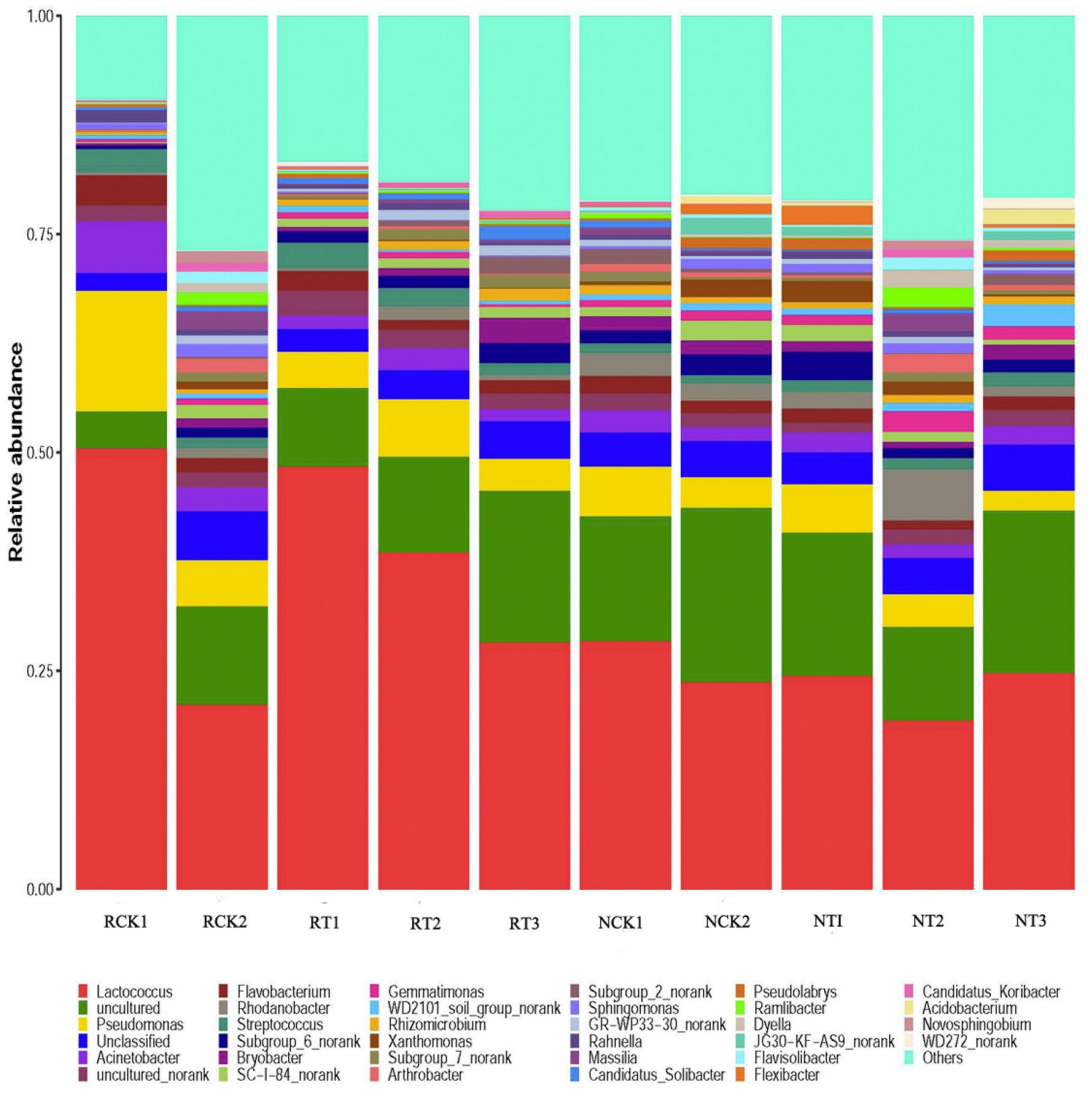
Relative read abundance of different bacterial community structures in different treatments at the genus level. RCK1: R-soil in CK1; RCK2: R-soil in CK2; RT1: R-soil in T1; RT2: R-soil in T2; RT3: R-soil in T3; NCK1: N-soil in CK1; NCK2: N-soil in CK2; NT1: N-soil in T1; NT2: N-soil in T2; NT3: N-soil in T3.

Further analysis investigated the relative bacterial community abundance at the genus level (Fig. 5). In total, 496 genera were recorded in all soil samples, among which *Lactococcus, uncultured, Pseudomonas, uncultured_norank, unclassified*, and *Flavobacterium* were the dominant genera. In N-soil samples, *Lactococcus* accounted for 23.76% of the total bacteria in CK2, and it accounted for comparatively higher proportion in T1 (24.51%) and T3 (24.82%), and a lower proportion in T2 treatment (19.41%). In R-soil samples, *Lactococcus* accounted for 21.13% of the total bacteria in CK2, and comparatively higher proportion in T1 (48.43%), T2 (38.64%), and T3 (28.34%).

**Figure 6:**
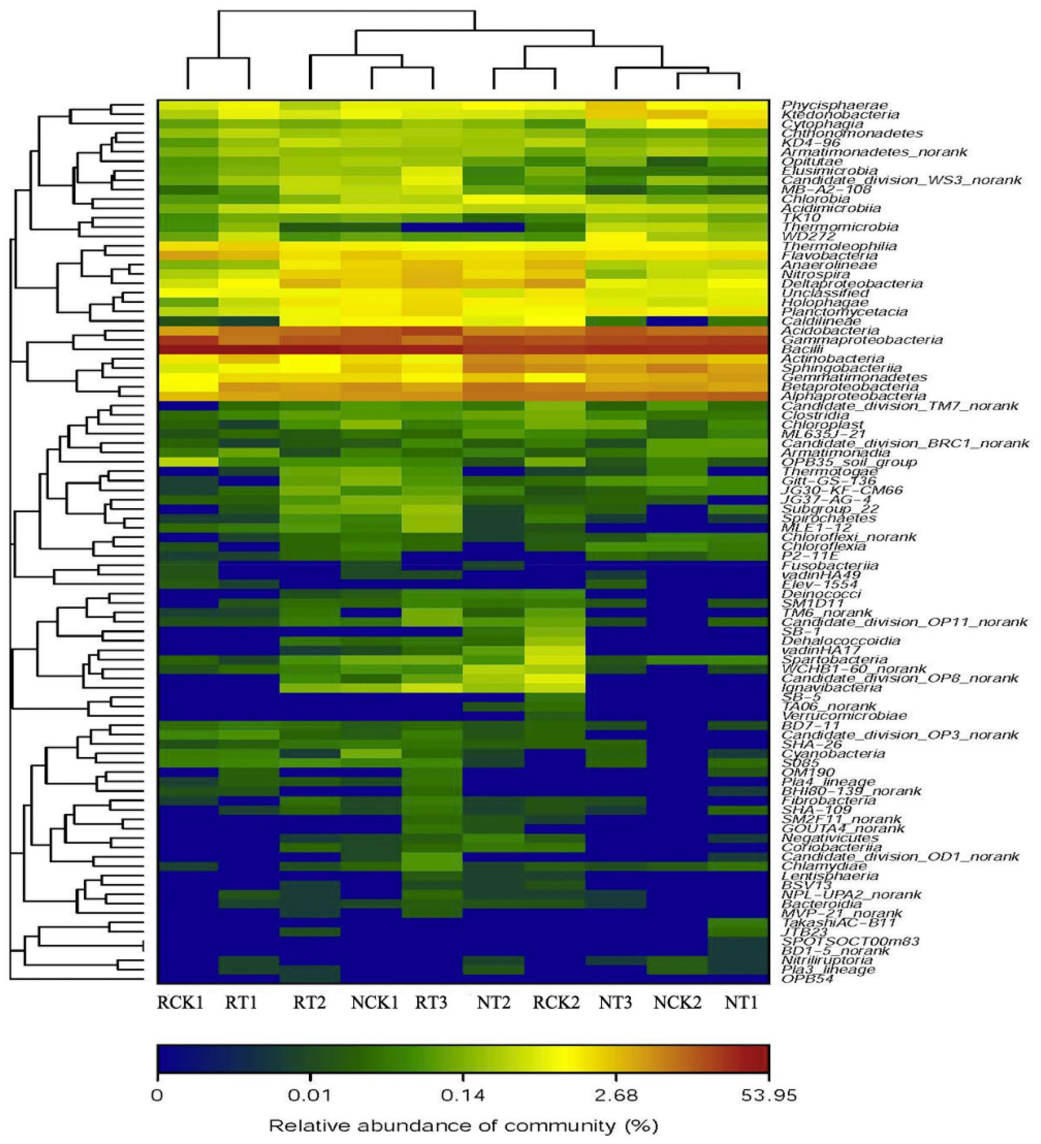
Species abundance clustering diagram of soil bacteria in different treatments at the genus level. RCK1: R-soil in CK1; RCK2: R-soil in CK2; RT1: R-soil in T1; RT2: R-soil in T2; RT3: R-soil in T3; NCK1: N-soil in CK1; NCK2: N-soil in CK2; NT1: N-soil in T1; NT2: N-soil in T2; NT3: N-soil in T3.

**Figure 7:**
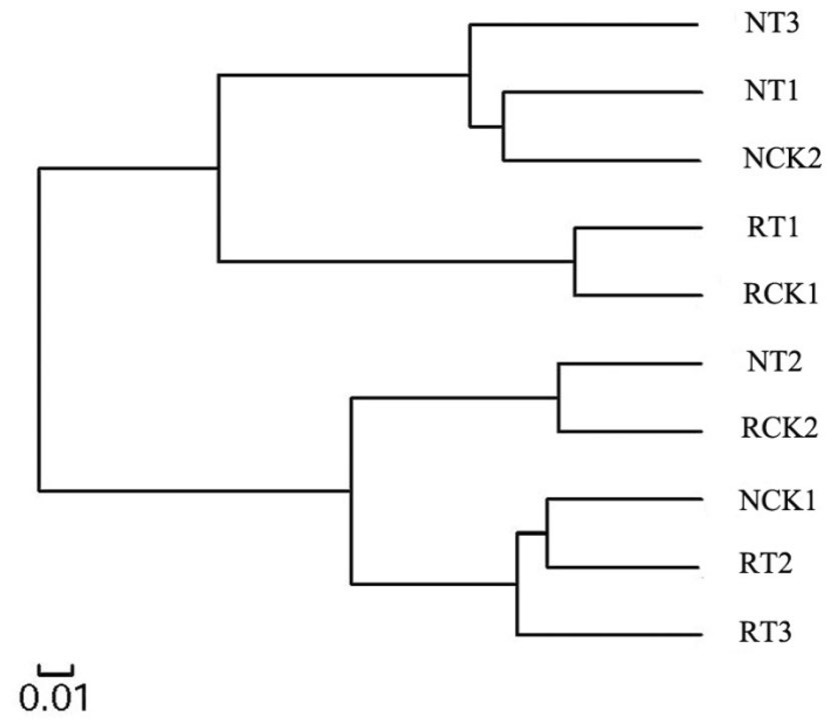
Clustering tree of soil bacteria in the different treatments. RCK1: R-soil in CK1; RCK2: R-soil in CK2; RT1: R-soil in T1; RT2: R-soil in T2; RT3: R-soil in T3; NCK1: N-soil in CK1; NCK2: N-soil in CK2; NT1: N-soil in T1; NT2: N-soil in T2; NT3: N-soil in T3.

The analysis of microbial community heatmap and similarity tree analysis for multiple samples identified the similarities and differences among the 10 bacterial community structures (Figs. 6, 7). The results showed that the communities were divided into two clusters based on similar community structure. CK2, T1, and T3 of N-soil were clustered together, whereas CK2, T2, and T3 of R-soil were clustered together, which also implied approximately similar bacterial community structure. And the high dose of biochar (50 g/kg) decreased the similarity of soil bacterial community structure in rhizosphere compared with those in non-rhizosphere soil. Additionally, the two clusters were well separated from each other, suggesting a clear distinction between the bacterial community structures.

## Discussion

The objective of this study was to evaluate the effect of straw biochar addition on tobacco growth, soil properties, and rhizosphere bacterial communities, which might be related to the dose effect of biochar. The obtained results suggested that biochar had an important effect on tobacco growth and a suitable dose of biochar (2–10 g/kg) could promote tobacco growth, but a higher dose (50 g/kg) could inhibit growth thus, supporting the results of previous studies^40–42^. Biochar application can improve soil pH and soil physicochemical properties^43,44^, and maximum nutrients can be desorbed from biochar and get readily absorbed into plants, which can subsequently promote tobacco growth. In the present study, our results showed that biochar addition increased few soil parameters, such as soil pH, soil organic carbon, and available nitrogen, phosphorus, and potassium. These results are consistent with the results of previous studies^45,46^. The improvement in soil nutrient contents by biochar addition is a direct impact of biochar, which can be attributed to its high nutritional value^47,48^. The application of biochar has other indirect influences on plant growth, such as increasing nutrient retention and nutrient use efficiency and reducing the nutrient leaching^49^. Therefore, biochar application could improve the overall crop growth by affecting nutrient uptake^50,51^. A recent meta-analysis study showed that variations in plant growth and productivity response had a pooled mean of 16.0 ± 1.3% and a range of − 31.8% to 974% under different biochar or/and soil conditions^52^.

In contrast, tobacco growth was inhibited by the addition of a high dose of biochar (40 mg/kg) in the current study (Table 1). There are also some reports of negative or insignificant effects of biochar on crop growth and yield. For example, Liu et al. (2013)^53^ found that crop productivity tended to decrease with increasing biochar application rates (> 40 t ha^−1^). Excessive amounts of biochar can inhibit crop growth and are related to N immobilization caused by high volatile content, toxic or harmful substance content, and reduced nutrient uptake and crop growth^54^. Heavy metals in the rice straw biochar were all below the threshold of heavy metals in biochar as reported by the International Biochar Initiative (IBI) and the European Biochar Certificate (EBC)^55^. The N immobilization maybe therefore be the limiting factor in tobacco growth. Nitrogen limitation may inhibit tobacco growth due to high doses of biochar, as the availability of N decreases because of N immobilization by microbial biomass at high C:N ratios, although other growth-limiting factors may be responsible for growth limitation as well^56,57^. The high dose of biochar application could improve the base cation content of K and soil pH (Table 2). The available K contributed most to the osmotic pressure and high pH which negatively affected plant growth^58^.

Soil enzyme activity is a key indicator of microbial function in nutrient retention and conversion associated with soil fertility. Previous studies have raised considerable doubts about the effect of biochar addition on the activity of soil hydrolases^18,59^, which may likely be associated with the biochar types and soil properties^60,61^. In this study, the addition of biochar increased the soil enzymatic activity of urease, invertase, and acid phosphatase, which was in accordance with the findings of previous studies^62,63^. Our results suggest that biochar might stimulate soil enzymic activity and affect bacterial community abundance via increasing soil organic carbon and available nitrogen, which is consistent with a previous study^64^. Large amounts of biochar addition (50 g/kg) significantly improved the SOC and available N contents (Tables 1, 3), which can be partly explained by the increase in the activity of invertase and urease. Furthermore, biochar has the capacity to absorb a variety of organic and inorganic molecules and inhibit certain soil enzymes or their substrates by adsorbing onto or blocking the reaction sites^17,18^, which may explain our results for catalase activities. Thus, the biochar amendment may contribute to maintaining or enhancing the enzymatic activity of C-, N-, and P-cycling in the soil.

According to Hammer et al. (2014)^65^, biochar presents a complex matrix for the diversity of soil microorganisms and the source of nutrients. In this study, biochar addition increased the N-soil bacterial diversity to a degree. Different doses of biochar showed different effects on soil bacterial abundance in this study. The medium biochar addition (10 g/kg, NT2) had a better effect on improving soil bacterial abundance, possibly because less (2 g/kg) or large (50 g/kg) amounts of biochar contains insufficient or excessive basic ions that are not suitable for soil microbial growth^54^. This result was consistent with the previous studies that lower doses of biochar had positive effects but higher doses of biochar caused toxicity and inhibition^66,67^. The biochar addition showed different effect on soil bacteria in rhizosphere in this study, that the richness of bacteria was increased, but the diversity decreased with the biochar addition (Table 4), which was consistent with the previous studies^68^. And the relative abundance of different bacterial community structures at the phylum or genus level (Figs. 3, 4) both convinced that the bacteria community in R-soil were sensitive to biochar addition than those in N-soil. The biochar nutrients, associated with biochar-influenced soil environment, can probably stimulate root growth (Table1) with the biochar addition. In turn, the carbon products secreted by facilitated growing roots, which are preferential nutrients and energy for microbial metabolism, can also affect microbial community in the rhizosphere soil^69^.

In the present study, the high-throughput sequencing analysis of Miseq indicated that biochar addition could significantly influence soil bacterial community structure either in rhizosphere or non-rhizosphere of tobacco (Table 4). Ace and Chao1 indices are indicators of microbial richness, whereas Shannon–Weiner and Simpson indices reflect microbial diversity^70^. Previous reports have shown that biochar may affect soil bacterial communities by improving the soil physico-chemical properties (e.g. sorption, pH, chemical properties, and habitats)^26,71,72^. Our results showed that biochar addition increased soil pH, organic carbon, and available soil nutrients. Besides, physical properties of biochar, such as high nanoporosity and large surface area could improve water retention and aeration in soil; thereby providing a better habitat for the growth of soil bacteria, which may lead to changes in the bacterial community structure^16,72^. The changes in chemical properties of soil after biochar addition may also explain the changes in bacterial communities^73^. Furthermore, the OTUs, as well as Ace, Chao1, Shannon–Weiner, and Simpson indices differed between rhizosphere and non-rhizosphere, and they differed with the different biochar doses. Rhizosphere is an important site for nutrient and energy exchange between plants and soil microbes, and the Root exudates are speculated to be the main driving force for growth of soil bacteria in different soils^74^. Plants can alter soil bacterial richness, diversity, structure, and function by modifying their root exudates^75^ Environmental changes were found to greatly affect the quantity and quality of root exudates^76^. These could be the reasons for the differences between rhizosphere and non-rhizosphere bacterial communities^77^.

In the case of the bacterial community composition, *Firmicutes, Proteobacteria, Acidobacteria, Bacteroidetes*, and *Actinobacteria* were the predominant (> 2%) phyla in all treatments (Fig. 3). *Proteobacteria* occupied the highest proportions (28.17–38.45%) of the bacterial sequences in N-soil, but *Firmicutes* occupied the highest proportions (23.39–53.98%) in R-soils. The proportion of *Firmicutes* was increased, but the proportions of *Proteobacteria* and *Bacteroidetes* were decreased in the R-soil compared with that in the N-soil under the biochar addition. This could be influenced by synergistic effects, such as co-metabolism or syntrophy of these bacteria, or because of their similar response patterns to biological, chemical, or physical variables, i.e., presence of a similar niche^78^. Taketani et al. (2013)^79^ found that the phylum *Proteobacteria*, which colonizes nutrient-rich environments, was the most predominant taxa in N-soil samples. It has been reported that *Actinobacteria* are often associated with the degradation of recalcitrant polymers, and thus, this phylum is considered ecologically important for decomposing organic matter in the soil. Meanwhile, at the genus level, the bacterial abundance was also distinct between R-soil and N-soil in biochar amended samples (Fig. 4). Furthermore, the PCoA result in this study showed significant differences in bacterial communities between the R-soil and N-soils despite the biochar addition, suggesting that the communities were highly influenced by the root exudates and rhizosphere environment. In previous studies, the analysis of the genetic relationships between the plants and the microbial communities showed that the bacterial compositions in the N-soils and R-soils were completely different, which might be a consequence of the action of tobacco root exudates^80,81^. Therefore, the effect of biochar on rhizosphere bacterial communities is a complex process. The inner mechanisms of the microorganisms still need elucidation through long-term experiments.

## Conclusions

Straw biochar application could affect tobacco agronomic traits and biomass to a certain degree. Low (2 g/kg) and moderate (10 g/kg) doses of biochar application had positive effects on tobacco agronomic traits, but growth was inhibited at high doses (50 g/kg). This capacity was associated with changes in soil pH, SOC, and related properties. Under biochar treatment, soil urease, invertase, and acid phosphatase activities were increased but catalase activity was decreased or remained unchanged with the increased biochar amendment.

Straw biochar addition had a significant effect on the bacterial community structure either in rhizosphere and non-rhizosphere soil. *Firmicutes, Proteobacteria, Acidobacteria, Bacteroidetes*, and *Actinobacteria* were the predominant (> 2%) phyla in all soil treatments. *Proteobacteria* occupied the highest proportions (28.17–38.45%) of the bacterial sequences in N-soil, but *Firmicutes* occupied the highest proportions (23.39–53.98%) in R-soils. Substantial differences in the bacterial communities between R-soil and N-soil were found despite the biochar addition.

In treatments with low and moderate dose of biochar, soil bacterial community structure of rhizosphere and non-rhizosphere soil, was similar to the control treatment soil. But high dose of biochar (50 g/kg) reduced such benefits especially in rhizosphere. So, the response of the bacterial community to straw biochar is a complex process affected by the biochar dosage and the root system, which would then drive the process of nutrient transformation and hence affect the growth of tobacco. However, the results of this study were based on a pot experiment over one growing season. In the future, long-term observations and field positioning experiments treated with straw biochar should be carried out to elucidate the effects of biochar on the soil micro-ecological environment, crop growth, and their inner mechanisms.
